# Hospital admission trends due to respiratory diseases in England and Wales between 1999 and 2019: an ecologic study

**DOI:** 10.1186/s12890-021-01736-8

**Published:** 2021-11-08

**Authors:** Abdallah Y. Naser, Munthir M. Mansour, Abeer F. R. Alanazi, Omar Sabha, Hassan Alwafi, Zahraa Jalal, Vibhu Paudyal, Mohammad S. Dairi, Emad M. Salawati, Jaber S. Alqahtan, Shalan Alaamri, Moaath K. Mustafa Ali

**Affiliations:** 1 Department of Applied Pharmaceutical Sciences and Clinical Pharmacy, Faculty of Pharmacy, Isra University, Amman, 11622 Jordan; 2 Department of Internal Medicine, University of Arkansas for Medical Center, Little Rock, AR 72205 USA; 3 Department of Pharmaceutical and Biological Sciences, UCL School of Pharmacy, London, UK; 4 Al Khalidi Hospital and Medical Center, Amman, 11183 Jordan; 5 Faculty of Medicine, Umm Al Qura University, Mecca, 21514 Saudi Arabia; 6 School of Pharmacy, Institute of Clinical Sciences, University of Birmingham, Birmingham, B15 2TT UK; 7 Family Medicine Department, Faculty of Medicine, King Abdulaziz University, Jeddah, Saudi Arabia; 8 Department of Respiratory Care, Prince Sultan Military College of Health Sciences, Dammam, Saudi Arabia; 9 Faculty of Medicine, Jeddah University, Jeddah, 24231 Saudi Arabia; 10 Greenebaum Comprehensive Cancer Center, University of Maryland, Baltimore, MD 20742 USA

**Keywords:** Admissions, England, Hospital, Respiratory, United Kingdom, Wales

## Abstract

**Background:**

Identifying trends of hospital admissions for respiratory diseases is crucial for public health and research to guide future clinical improvements for better outcomes. This study aims to define the trends of respiratory disease-related hospital admissions (RRHA) in England and Wales between 1999 and 2019.

**Methods:**

An ecological study was conducted using hospital admission data taken from the Hospital Episode Statistics database in England and the Patient Episode Database for Wales. Hospital admissions data for respiratory diseases were extracted for the period between April 1999 and March 2019. The trend in hospital admissions was assessed using a Poisson model.

**Results:**

Hospital admission rate increased by 104.7% [from 1535.05 (95% CI 1531.71–1538.38) in 1999 to 3142.83 (95% CI 3138.39–3147.26) in 2019 per 100,000 persons, trend test, *p* < 0.01]. The most common causes were influenza and pneumonia, chronic lower respiratory diseases, other acute lower respiratory infections, which accounted for 26.6%, 26.4%, and 14.9%, respectively. The age group 75 years and above accounted for 34.1% of the total number of hospital admissions. Males contributed to 50.5% of the total number of hospital admissions. Hospital admission rate in females increased by 119.8% [from 1442.18 (95% CI 1437.66–1446.70) in 1999 to 3169.38 (95% CI 3163.11–3175.64) in 2019 per 100,000 persons, trend test, *p* < 0.001]. Hospital admission rate increased by 92.9% in males [from 1633.25 (95% CI 1628.32–1638.17) in 1999 to 3149.78 (95% CI 3143.46–3156.09) in 2019 per 100,000 persons, trend test, *p* < 0.001].

**Conclusion:**

During the study period, hospital admissions rate due to respiratory diseases increased sharply. The rates of hospital admissions were higher among males for the vast majority of respiratory diseases. Further observational studies are warranted to identify risk factors for these hospital admissions and to offer relevant interventions to mitigate the risk.

## Background

With each breath, the human lung is constantly exposed to airborne pollutants, irritants and infectious agents, making respiratory diseases one of the leading causes of morbidity and death globally [1–3]. In the United Kingdom (UK), respiratory diseases account for 6.5% of hospital admissions and 24% of all deaths [2–4]. These diseases impair the quality of life of the patients and have a negative impact on societies [5, 6]. Nowadays, respiratory diseases particularly respiratory tract infections, continue to impose an immense worldwide health burden [7]. Over the decades, many factors influenced the staggering incidence rates of respiratory diseases [7]. In high-income countries, tobacco smoke, indoor air pollution from burning fuels, low socioeconomic status, air pollution from traffic and industrial sources are important contributors to most respiratory conditions [7, 8].

Chronic obstructive pulmonary disease (COPD), reactive airway diseases, lower and upper respiratory tract infections, occupational lung diseases and lung cancer are some of the leading causes of hospital admissions due to respiratory diseases in the UK, and they constitute serious public health problems [9, 10]. Additionally, respiratory infections are one of the leading causes of death in developing countries despite the significant advancements in antibiotics. The Global Burden of Disease Group has estimated that lower respiratory tract infections alone cause 2.74 million deaths while three million die from COPD [11]. No research was previously conducted to investigate the trends of hospital admissions due to respiratory diseases in the UK in the past two decades. Therefore, this study sought to define the trends of respiratory disease-related hospital admissions (RRHA) in England and Wales between 1999 and 2019 through conducting a comprehensive analysis using publicly available data.

## Methods

### Data sources and study population

As previously described [12–14], we conducted an ecological study using publicly available data from the two central medical databases in England and Wales; the Hospital Episode Statistics (HES) database in England and the Patient Episode Database for Wales (PEDW). The HES database provides detailed information on hospital admission associated with a wide range of health conditions in England and the PEDW provides similar information related to Wales residents.

Hospital admission data were collected for the period between April 1999 and March 2019. The HES and PEDW databases contain hospital admission data for all types of diseases for respiratory system related (DRS) for patients from all age categories, including below 15 years, 15–59 years, 60–74 years, and 75 years and above. We identified DRS admission using the tenth version of the International Statistical Classification of Diseases (ICD) system. All diagnostic codes for diseases of the respiratory system (J00–J99) were used to identify all hospital admission related to various types of diseases of the respiratory system in England and Wales. The HES and PEDW databases record all hospital admissions, outpatient visits and accident and emergency attendances performed at all National Health Service (NHS) trusts and any independent sector funded by NHS trusts. Data for hospital admissions in England and Wales are available from the years 1999/2000 onwards. Available data include patient demographics, clinical diagnoses, procedures, and duration of stay. The HES and PEDW data are checked regularly to ensure their validity and accuracy. To calculate the annual hospital admission rate for DRS, we collected mid-year population data between 1999 and 2019 from the Office for National Statistics.

### Data analysis

Annual DRS admission rates with 95% confidence intervals (CIs) were calculated using the number of hospital admissions related to each type of respiratory disease for each age group divided by the mid-year population of the same age group of the same year. The Chi-square test was used to assess the difference between the hospital admission rates in 1999 and 2019. The trend in hospital admissions was assessed using a Poisson model. We conducted three independent Poisson regression models included the DRS rate as the dependent variable, while the independent variable for the three models were the years of admission for the overall population, gender (across the years), and the age group (across the years) as available data from the two databases were stratified in this manner. A two-sided *p* < 0.05 was considered statistically significant. All analyses were performed using SPSS version 25 (IBM Corp, Armonk, NY, USA).

## Results

The total annual number for RRHA for diverse causes increased by 133.4% from 800,374 in 1999 to 1,868,092 in 2019, representing an increase in hospital admission rate of 104.7% [from 1535.05 (95% CI 1531.71–1538.38) in 1999 to 3142.83 (95% CI 3138.39–3147.26) in 2019 per 100,000 persons, trend test, *p* < 0.01].

During the study period, the most prevalent diseases of the RRHA culprits were influenza and pneumonia, chronic lower respiratory diseases, other acute lower respiratory infections, acute upper respiratory infections, and other diseases of upper respiratory tract, which accounted for 26.6%, 26.4%, 14.9%, 11.3%, and 10.1%, respectively (Table 1).

**Table 1:** Percentage of diseases of the respiratory system hospital admission from the total number of admissions per ICD code during the study period

ICD code	Description	Percentage from the total number of admissions (%)
J00-J06	Acute upper respiratory infections	11.3
J09-J18	Influenza and pneumonia	26.6
J20-J22	Other acute lower respiratory infections (Acute bronchitis, Acute bronchiolitis, and Unspecified acute lower respiratory infection)	14.9
J30-J39	Other diseases of upper respiratory tract (Vasomotor and allergic rhinitis, Chronic rhinitis, nasopharyngitis and pharyngitis, Chronic sinusitis, Nasal polyp, Other and unspecified disorders of nose and nasal sinuses, Chronic diseases of tonsils and adenoids, Peritonsillar abscess, Chronic laryngitis and laryngotracheitis, Diseases of vocal cords and larynx, not elsewhere classified, and Other diseases of upper respiratory tract))	10.1
J40-J47	Chronic lower respiratory diseases	26.4
J60-J70	Lung diseases due to external agents	2.5
J80-J84	Other respiratory diseases principally affecting the interstitium (Acute respiratory distress syndrome, Pulmonary edema, Pulmonary eosinophilia, not elsewhere classified, and Other interstitial pulmonary diseases)	1.5
J85-J86	Suppurative and necrotic conditions of the lower respiratory tract	0.5
J90-J94	Other diseases of the pleura (Pleural effusion, not elsewhere classified, Pleural effusion in conditions classified elsewhere, Pleural plaque, Pneumothorax and air leak, and Other pleural conditions))	3.9
J95-J95	Intraoperative and postprocedural complications and disorders of respiratory system, not elsewhere classified	0.2
J96-J99	Other diseases of the respiratory system (Acute respiratory distress syndrome, Pulmonary edema, Pulmonary eosinophilia, not elsewhere classified, and Other interstitial pulmonary diseases)	2.1

ICD, International Statistical Classification of Diseases system

**Fig. 1:**
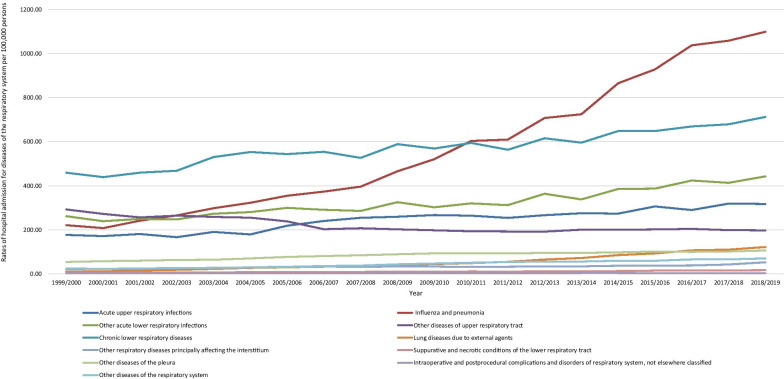
Hospital admission rates due to diseases of the respiratory system in England and Wales stratified by type between 1999 and 2019

During the past two decades, the largest increase in the rate of hospitalisation was noted in lung diseases due to external agents, followed by influenza and pneumonia, other diseases of the respiratory system, suppurative and necrotic conditions of the lower respiratory tract, and other respiratory diseases principally affecting the interstitium with 10.5-fold, 4.0-fold, 1.8-fold, 1.5-fold, and 1.4-fold, respectively. Furthermore, the rate of hospitalisation due to other diseases of the pleura, acute upper respiratory infections, other acute lower respiratory infections, chronic lower respiratory diseases, and intraoperative and postprocedural complications and disorders of respiratory system, not elsewhere classified was increased by 95.2%, 79.2%, 68.9%, 55.1%, and 20.8%, respectively. However, the rate of hospital admission due to other diseases of upper respiratory tract decreased by 32.4% (Fig. 1).

**Fig. 2:**
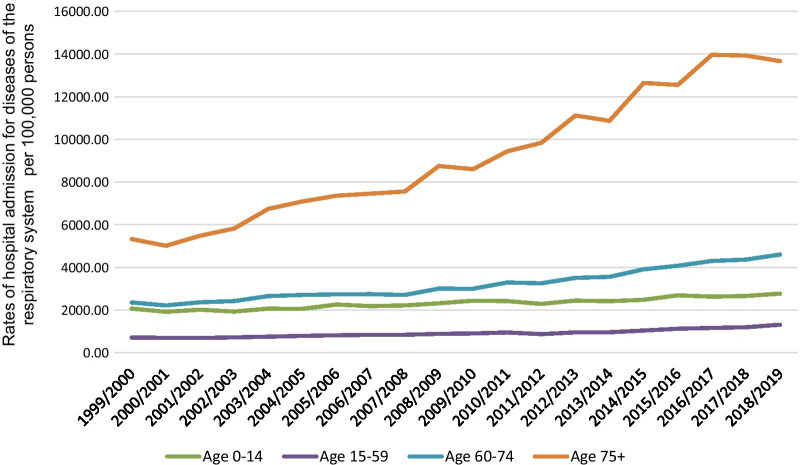
Rates of hospital admission for all diseases of the respiratory system in England and Wales stratified by age group

Regarding RRHA distribution among different age groups, the age group 75 years and above accounted for 34.1% of the total number of RRHA, followed by 15–59 years age group with 25.2%, the age group 60–74 years with 21.6%, and then the age group below 15 years with 19.2%. The RRHA rate among patients aged below 15 years increased by 34.0% [from 2068.49 (95%CI 2059.63–2077.35) in 1999 to 2771.04 (95%CI 2761.21–2780.86) in 2019 per 100,000 persons, trend test, *p* < 0.001]. The RRHA rate increased by 84.2% among patients aged 15–59 years [from 712.37 (95%CI 709.43–715.31) in 1999 to 1312.11 (95%CI 1308.30–1315.91) in 2019 per 100,000 persons]. The RRHA rate increased by 95.6% among patients aged 60–74 years [from 2353.38 (95%CI 2342.10–2364.67) in 1999 to 4603.87 (95%CI 4590.36–4617.38) in 2019 per 100,000 persons, trend test, *p* < 0.001]. The RRHA rate increased by 156.5% [from 5329.72 among patients aged 75 years and above (95%CI 5307.47–5351.97) in 1999 to 13,668.22 (95%CI 13,638.34–13,698.10) in 2019 per 100,000 persons, trend test, *p* < 0.001] (Fig. 2).

**Fig. 3:**
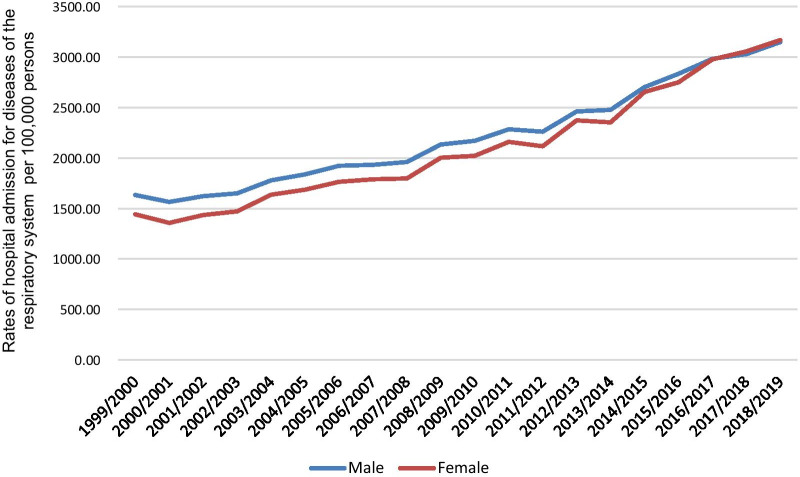
Rates of hospital admission for all diseases of the respiratory system in England and Wales stratified by gender

A total of 24,202,487 RRHA episodes were reported in England and Wales during the study duration. Males contributed to 50.5% of the total number of diseases of the RRHA accounting for 12,234,065 hospital admission episodes by a mean of 611,703 per year. The RRHA rate in females increased by 119.8% [from 1442.18 (95% CI 1437.66–1446.70) in 1999 to 3169.38 (95% CI 3163.11–3175.64) in 2019 per 100,000 persons, trend test, *p* < 0.001]. The RRHA rate increased by 92.9% in males [from 1633.25 (95% CI 1628.32–1638.17) in 1999 to 3149.78 (95% CI 3143.46–3156.09) in 2019 per 100,000 persons, trend test, *p* < 0.001] (Fig. 3).

### Diseases of the respiratory system hospital admission rate by gender

**Fig. 4:**
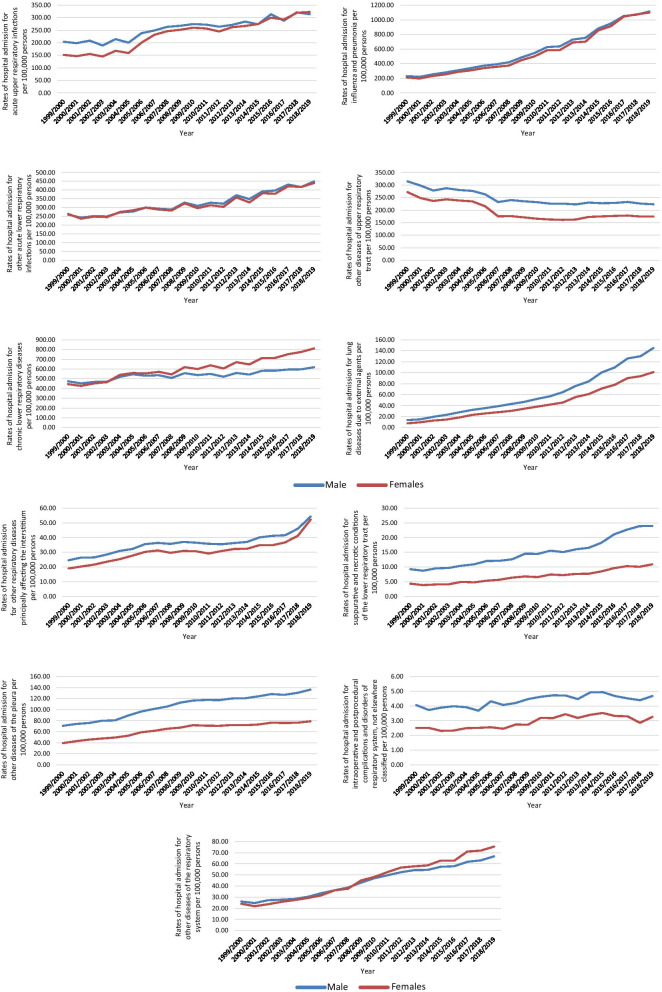
Hospital admission rates for diseases of the respiratory system in England and Wales stratified by gender

The rates of RRHA were higher among males compared to females except for chronic lower respiratory diseases and other diseases of the respiratory system, which were more common across females (*p* < 0.05) (Fig. 4).

### Diseases of the respiratory system hospital admission rate by age group

**Fig. 5:**
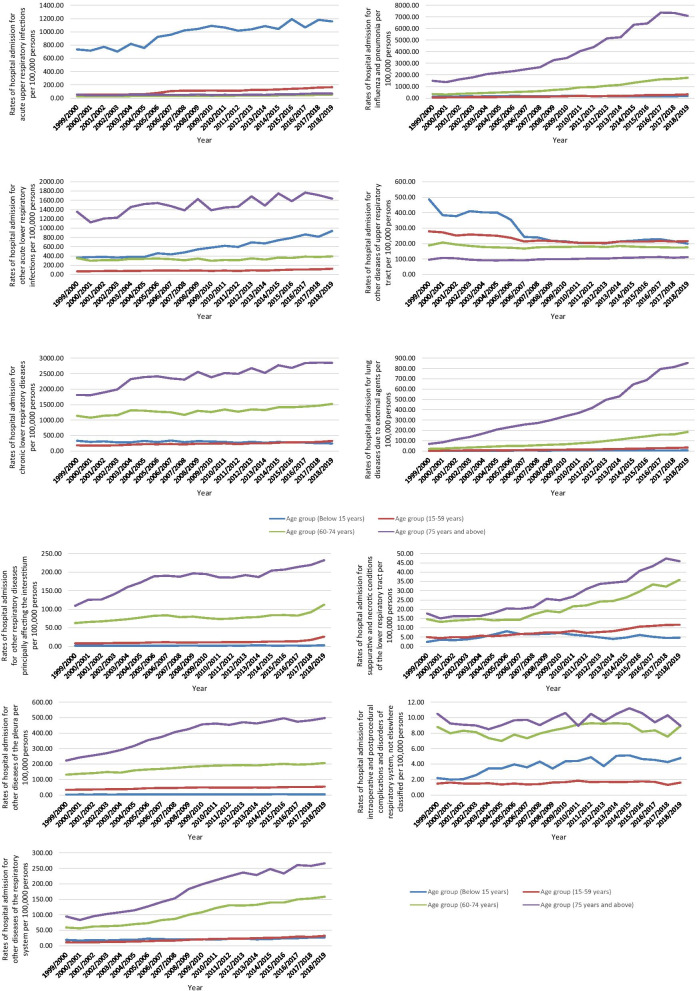
Hospital admission rates for diseases of the respiratory system in England and Wales stratified by age group

Hospital admissions secondary to diseases of the respiratory system varied with age groups. That included influenza and pneumonia, lung diseases due to external agents, other respiratory diseases principally affecting the interstitium, suppurative and necrotic conditions of the lower respiratory tract, and other pleura diseases. However, hospital admissions due to chronic lower respiratory diseases, intraoperative and postprocedural complications and disorders of respiratory system, not elsewhere classified, and other diseases of the respiratory system were common among the age group: 75 years and above, 60–74 years, below 15 years, and 15–59 years, respectively. Hospital admissions due to acute upper respiratory infections were higher among the age group: below 15 years, 15–59 years, 75 years and above, and 60–74 years, respectively. Hospital admissions due to other acute lower respiratory infections were common among the age group: 75 years and above, below 15 years, 60–74 years, and 15–59 years, respectively. Nevertheless, other diseases of upper respiratory tract hospital admission rate were inversely related to age, with a higher incidence in the age group below 15 years (Fig. 5).

## Discussion

The global death toll of lung disease is tremendous, with three respiratory diseases: COPD, lower respiratory tract infections, and diseases of the trachea, bronchus and lung cancer, ranking among the leading ten causes of death worldwide [15]. Similar to other countries, respiratory diseases are a significant cause of morbidity, mortality, and financial burden in the UK [2]. In this study, we found that RRHA has increased by 133.4% between 1999 and 2019 in England and Wales. The increase in hospital admissions can not be explained solely by the absolute increase in the population size, which increased by approximately 14% between 1999 and 2019 in the UK, or the increase in migration which mainly concerns the younger age group [16, 17]. The life expectancy continues to increase in the UK and is estimated to be 79.4 and 83.1 years for males and females born between 2017 and 2019, respectively [18]. As the UK population is aging, the elderly pool at higher risk for hospitalization expands. Moreover, the age group above 65 is the fastest-growing age category in the UK [19]. Congruent with our analysis, we showed that the rate of RRHA increases with aging. Figure 2 demonstrates that the fastest increase in RRHA was seen age category 75 years and older. In 2050, it is projected that 25% of the population of the UK will be above 65 years [19]. Aging is associated with significant changes in lung function, structure and waning immunity, resulting in increased risk for morbidity and mortality secondary to respiratory diseases [20].

Another factor that may explain the high RRHA is smoking, which is a strong risk factor for many respiratory diseases. Despite that smoking cigarettes prevalence continues to drop in the UK, around 14% of adults aged 18 and above are known to be current smokers. It is important to note that the effect of decreased smoking prevalence on public health may take many years to be noticed due to the chronicity of most smoking related diseases [18, 21]. Exposure to second-hand smoke can precipitate a range of respiratory diseases, many of which are fatal, especially in children and vulnerable individuals [22]. From 2018 to 2019 alone, there were more than 500,000 hospital admissions attributable to smoking in England. Reducing the prevalence of cigarette smoking, therefore, should be a primary objective for the government (17). Electronic cigarette (EC) use was introduced in 2007 as a potential solution to combat smoking health-related conditions. However, several studies highlighted the potential risk of EC on the cellular and organ level [23, 24]. Madison et al. [25] demonstrated that chronic e-cigarette vapor aberrantly alters the physiology of lung epithelial cells and innate immunity, increasing risk of recurrent pneumonias and thus COPD. Nevertheless, the use of EC is still increasing particularly in younger patients. Because EC’s public health impact in the UK and worldwide is not well established, more research in the field is needed.

Air pollution is another factor that can explain the increase in RRHA. Exposure to air pollution can reduce lung function, increase the risk for respiratory diseases and cause asthma and COPD exacerbation [26]. The European Environmental Agency considered air pollution as the most considerable environmental health hazard in Europe [26]. It was estimated that 40,000 premature deaths have occurred from air pollution in the UK [26]. The prevalence of asthma increased from 5.4% and 5.8% in men and women respectively in 1972 to 15.4% and 20.0% in 1996; this can be attributed to the increment in air pollution due to traffic and industrial sources [27]. The impact of air pollution on respiratory health is expected to increase in the following decades due to increasing population size and global warming, and climate change.

Notably, the rates of RRHA from ICD categories, acute upper respiratory infections and other acute lower respiratory infections were higher in younger age groups. On average, youngest children contract 6–8 upper respiratory tract infections yearly compared to 2–4 per year in adults [28]. The inverse relation between the incidence of upper respiratory tract infections and age is the likely cause of the finding in our study [28]. Influenza and pneumonia account for more than one-quarter of hospital admissions in England and the UK. The finding is not unexpected, as lower respiratory tract infections are the number four leading cause of mortality globally [15]. Vaccination programs were proven to be highly effective in preventing respiratory infections, including influenza and bacterial pneumonia [29].

Nevertheless, we lack vaccines against many important respiratory pathogens, such as the respiratory syncytial virus, which can cause severe fatal pneumonia in individuals at risk [30]. Unfortunately, breakthrough infections in vaccinated patients happen. In addition, many viruses can mutate, leading to more virulent strains that can spread more quickly and cause severe disease [31]. Management of respiratory infections has improved over the last two decades, but the battle against infectious pathogens continues.

The ICD category chronic lower respiratory diseases including COPD, asthma, and bronchiectasis, accounts for almost one-quarter of RRHA in England and Wales. The British Lung Foundation estimated that 1.2 million patients are diagnosed with COPD in 2012 [32]. In 2018, chronic lower respiratory diseases were the fourth leading cause of death in the UK and accounted for 17,988 deaths [33]. Furthermore, COPD’s prevalence and associated mortality is projected to increase by 39% and 30% between 2011 and 2030 in England, respectively [34]. The cost impact of COPD on the UK economy and health system is considerable, and it is estimated that 24 million working days are lost each year due to COPD [35]. The primary determinant of health burden from COPD is inpatient hospitalization [36], suggesting that reducing the patient need for hospital care could minimize the burden of COPD on the UK health system. This can be achieved by ensuring earlier diagnosis and use of interventions to prevent exacerbations and delay the progression of the disease [35–37]. Such prevention strategies were found as top-ten research priorities in a joint patient-clinician research prioritising exercise for exacerbations of COPD [38].

Over the last two decades, the largest increase in RRHA rate was seen in ICD category of lung diseases due to external agents, which include occupational lung diseases, respiratory conditions due to inhalation of chemicals, gases, fumes and vapors and pneumonitis due to solids and liquids. In a 2018 report by the Irish Thoracic Society, 95% of admissions from this category were attributed to pneumonitis due to solids and liquids category [39]. Inhalation of food and vomit, also known as aspiration pneumonia, has many risk factors, including decreased consciousness, mechanical ventilation, poor oral hygiene and aging [40]. Figure 5 shows that the fastest increase in RRHA rate in the ICD category lung diseases due to external agents were seen in the age category 75 years and older. Our study did not show a significant difference in RRHA between males and females. However, the increase in the RRHA rate was higher in females (119.8%) than males (92.9%). This could be explained partially by females’ more extended life expectancy, which increases the pool of patients susceptible for RRHA [19]. As the socio-occupational disparities dissipate between males and females, females are contracting an increasing number of respiratory diseases worldwide [41]. In 2000, more females died from COPD than men globally, reflecting the dynamic change in disease prevalence and incidence [41]. Figure 4 shows that RRHA due to lower respiratory disease that includes COPD is higher in females than males, with an increasing gap over the years.

During COVID-19 pandemic, there was a 50% reduction in COPD admissions compared to the pre-COVID-19 years and this was associated with a decline in respiratory viral infections that trigger exacerbations [42]. Another study conducted in the UK found a significant decrease in asthma exacerbations during the COVID-19 pandemic [43]. This reduction was also seen in bronchiectasis patients compared to the same times in the previous 2 years before the COVID-19 pandemic [44]. This might be explained by the impact of coronavirus preventive measures on RRHA. However, this reduction in hospitalisation could be driven by the reluctance of patients to be admitted to a hospital during the COVID-19 pandemic [45]. As of now, there are no recommendations in the respiratory diseases guidelines for infection control measures against respiratory viruses that lead to respiratory admissions. The inclusion of such measures such as hand hygiene and face coverings might potentially lower the burden of admissions for respiratory diseases beyond COVID-19 pandemic.

This study has clinical and research implications. Firstly, the ability to control respiratory diseases relies on public health measures, including increasing awareness and knowledge about the burden of respiratory diseases and incorporating simple infection control measures in the future guidelines for respiratory diseases. Secondly, air pollution and cigarette smoking remain the two major preventable culprits for respiratory diseases, and the government should continue to fund research related to both to help alleviate the financial burden of RRHA in the following decades. Thirdly, for chronic respiratory diseases, early diagnosis is essential to decrease RRHA. Moreover, sufficient investment in proven therapies could prevent patients with chronic respiratory conditions from needing hospital admissions, like pulmonary rehabilitation and smoking cessation. Finally, proactive research is needed to improve our understanding of the risk factors and comorbidities associated with admissions due to respiratory diseases.

## Conclusion

This study revealed that RRHA increased by 133.4% in the last two decades in England and Wales, in which men were more likely to be hospitalized.Respiratory diseases can be preventable, and the associated prevention costs are only a fraction of treatment costs. Future research with innovative approaches are needed to lessen the clinical burden of these hospital admissions, enabling disease prevention, timely diagnosis and new effective personalized interventions.

## Data Availability

The datasets used and/or analysed during the current study are available from the corresponding author on reasonable request.

## Abbreviations

RRHA: Respiratory disease related hospital admission
UK: United Kingdom
COPD: Chronic obstructive pulmonary disease
HES: Hopital episode statistcis
PEDW: Patient episode statistcs for Wales
SPSS: Statistical package for social sciences
ICD: International claissifcation of diseases
DRS: Diseases for respiratory system
CI: Confidence interval

## References

[1] Burney P, Jarvis D, Perez-Padilla R (2015). The global burden of chronic respiratory disease in adults. Int J Tuberc Lung Dis.

[2] Chung F (2002). Assessing the burden of respiratory disease in the UK. Respir Med.

[3] Hubbard R (2006). The burden of lung disease. Thorax.

[4] Lozano R (2012). Global and regional mortality from 235 causes of death for 20 age groups in 1990 and 2010: a systematic analysis for the Global Burden of Disease Study 2010. Lancet.

[5] Boot CR, van Exel NJ, van der Gulden JW (2009). "My lung disease won't go away, it's there to stay": profiles of adaptation to functional limitations in workers with asthma and COPD. J Occup Rehabil.

[6] Eisner MD (2002). The influence of chronic respiratory conditions on health status and work disability. Am J Public Health.

[7] Troeger C, Blacker B, Khalil IA, Rao PC, Cao J, Zimsen SR, Albertson SB, Deshpande A, Farag T, Abebe Z, Adetifa IM (2018). Estimates of the global, regional, and national morbidity, mortality, and aetiologies of lower respiratory infections in 195 countries, 1990–2016: a systematic analysis for the Global Burden of Disease Study 2016. Lancet Infect Dis.

[8] Forum of International Respiratory Societies (2017). The global impact of respiratory disease.

[9] Salciccioli JD (2018). Respiratory disease mortality in the United Kingdom compared with EU15+ countries in 1985–2015: observational study. BMJ.

[10] Upton MN (2000). Intergenerational 20 year trends in the prevalence of asthma and hay fever in adults: the Midspan family study surveys of parents and offspring. BMJ.

[11] Lim SS (2012). A comparative risk assessment of burden of disease and injury attributable to 67 risk factors and risk factor clusters in 21 regions, 1990–2010: a systematic analysis for the Global Burden of Disease Study 2010. Lancet.

[12] Naser AY, Alrawashdeh HM, Alwafi H, AbuAlhommos AK, Jalal Z, Paudyal V, Alsairafi ZK, Salawati EM, Samannodi M, Sweiss K, Aldalameh Y, Alsaleh FM, Abusamak M, Shamieh A, Tantawi EI, Dairi MS, Dairi M. Hospital admission trends due to viral infections characterised by skin and mucous membrane lesions in the past two decades in England and Wales: an ecological study. Int J Environ Res. Public Health. 2021;18:11649. 10.3390/ijerph182111649. 10.3390/ijerph182111649 PMC8582963 34770162

[13] Hemmo SI (2021). Hospital admissions due to ischemic heart diseases and prescriptions of cardiovascular diseases medications in England and Wales in the past two decades. Int J Environ Res Public Health.

[14] Naser AY (2018). Hospital admissions due to dysglycaemia and prescriptions of antidiabetic medications in England and Wales: an ecological study. Diabetes Ther.

[15] WHO. The top 10 causes of death. 2020. https://www.who.int/news-room/fact-sheets/detail/the-top-10-causes-of-death. Accessed 7 Dec 2021.

[16] Office for National Statistics. Population estimates. https://www.ons.gov.uk/peoplepopulationandcommunity/populationandmigration/populationestimates. Accessed 7 Dec 2021.

[17] Office for National Statistics. Revised population estimates for England and Wales: mid-2012 to mid-2016. 2018. https://www.ons.gov.uk/peoplepopulationandcommunity/populationandmigration/populationestimates/bulletins/annualmidyearpopulationestimates/mid2012tomid2016. Accessed 31 Oct 2021.

[18] Office for National Statistics. Adult smoking habits in the UK: 2019. 2019. https://www.ons.gov.uk/peoplepopulationandcommunity/healthandsocialcare/healthandlifeexpectancies/bulletins/adultsmokinghabitsingreatbritain/2019. Accessed 7 Dec 2021.

[19] Office for National Statistics. Overview of the UK population: January 2021. 2021. https://www.ons.gov.uk/peoplepopulationandcommunity/populationandmigration/populationestimates/articles/overviewoftheukpopulation/january2021. Accessed 7 Dec 2021

[20] Meyer KC (2005). Aging. Proc Am Thorac Soc.

[21] Bonnie RJ, Stratton K, Kwan LY (2015). Public health implications of raising the minimum age of legal access to tobacco products.

[22] Carreras G (2019). Burden of disease attributable to second-hand smoke exposure: a systematic review. Prev Med.

[23] Cunningham TJ (2015). Associations of self-reported cigarette smoking with chronic obstructive pulmonary disease and co-morbid chronic conditions in the United States. COPD.

[24] Bowler RP (2017). Electronic cigarette use in US adults at risk for or with COPD: analysis from two observational cohorts. J Gen Intern Med.

[25] Madison MC (2019). Electronic cigarettes disrupt lung lipid homeostasis and innate immunity independent of nicotine. J Clin Invest.

[26] European Environmet Agency. *EEA Report No 9/2020*. 2020. https://www.eea.europa.eu/publications/air-quality-in-europe-2020-report.

[27] Guarnieri M, Balmes JR (2014). Outdoor air pollution and asthma. Lancet.

[28] Heikkinen T, Järvinen A (2003). The common cold. Lancet (London, England).

[29] Domínguez A (2013). Effectiveness of vaccination with 23-valent pneumococcal polysaccharide vaccine in preventing hospitalization with laboratory confirmed influenza during the 2009–2010 and 2010–2011 seasons. Hum Vaccin Immunother.

[30] Fraser CS, Jha A, Openshaw PJ (2017). vaccines in the prevention of viral pneumonia. Clin Chest Med.

[31] Menachery VD (2015). A SARS-like cluster of circulating bat coronaviruses shows potential for human emergence. Nat Med.

[32] British Lung Foundation. Chronic obstructive pulmonary disease (COPD) statistics. 2012. https://statistics.blf.org.uk/copd?_ga=2.219875801.1367299004.1527163268-1758129798.1527163268. Accessed 7 Dec 2021.

[33] Office for National Statistics. Leading causes of death, UK: 2001 to 2018. 2018. https://www.ons.gov.uk/peoplepopulationandcommunity/healthandsocialcare/causesofdeath/articles/leadingcausesofdeathuk/2001to2018. Accessed 7 Dec 2021.

[34] McLean S (2016). Projecting the COPD population and costs in England and Scotland: 2011 to 2030. Sci Rep.

[35] Lopez AD (2006). Chronic obstructive pulmonary disease: current burden and future projections. Eur Respir J.

[36] Britton M (2003). The burden of COPD in the U.K.: results from the Confronting COPD survey. Respir Med.

[37] Ferkol T, Schraufnagel D (2014). The global burden of respiratory disease. Ann Am Thorac Soc.

[38] Alqahtani JS (2021). Research priorities for exacerbations of COPD. Lancet Respir Med.

[39] Irish Thoracic Society. Respiratory Diseases Due to External Agents. 2018. https://irishthoracicsociety.com/wp-content/uploads/2019/04/Chapter-11-Respiratory-Diseases-due-to-External-Agents.pdf. Accessed 7 Dec 2021.

[40] Marik PE (2001). Aspiration pneumonitis and aspiration pneumonia. N Engl J Med.

[41] Pinkerton KE (2015). Women and lung disease. Sex differences and global health disparities. Am J Respir Crit Care Med.

[42] Alqahtani JS (2021). Reduction in hospitalised COPD exacerbations during COVID-19: A systematic review and meta-analysis. PLoS ONE.

[43] Shah SA (2021). Impact of COVID-19 national lockdown on asthma exacerbations: interrupted time-series analysis of English primary care data. Thorax.

[44] Crichton ML, Shoemark A, Chalmers JD (2021). The impact of the COVID-19 pandemic on exacerbations and symptoms in bronchiectasis: a prospective study. Am J Respir Crit Care Med..

[45] Tan J (2021). COVID-19 public health measures: a reduction in hospital admissions for COPD exacerbations. Thorax.

